# Living with systemic lupus erythematosus in South Africa: a bitter pill to swallow

**DOI:** 10.1186/s12955-019-1132-y

**Published:** 2019-04-16

**Authors:** A. Phuti, M. Schneider, K. Makan, M. Tikly, B. Hodkinson

**Affiliations:** 1 Rheumatic Disease Unit, Department of Medicine, Groote Schuur Hospital, University of Cape Town, Cape Town, South Africa; 2 Alan J Flisher Centre for Public Mental Health, University of Cape Town, Cape Town, South Africa; 3 Division of Rheumatology, Department of Medicine, Chris Hani Baragwanath Academic Hospital, University of the Witwatersrand, Johannesburg, South Africa

**Keywords:** Systemic lupus erythematosus, Experiences, Perceptions, Health related quality of life, Qualitative, Africa

## Abstract

**Background:**

Systemic lupus erythematosus (SLE) often has a profound negative impact on health-related quality of life (HRQoL). In the absence of any qualitative studies in sub-Saharan Africa, we undertook a study to explore living experiences, perceptions and unmet needs of South African patients with SLE.

**Methods:**

Twenty-five women with SLE consented to participate in the study. They underwent individual in-depth interviews exploring their physical concerns, emotional health, sexual well-being and fertility. NVivo software was used for analysis.

**Results:**

Participants were either of black ancestry or mixed racial ancestry, mainly indigent with only a quarter gainfully employed. Living with pain was the most common complaint, negatively impacting on activities of daily living (ADL), family expectations, social life, sleep and intimacy. Most participants expressed challenges of living with fatigue, and many felt their fatigue was misconstrued as being ‘simply lazy’. This pernicious fatigue had negative consequences on many facets of ADL, including caring for dependants, job sustainability and sexual well-being. All participants experienced low emotional states, often associated with suicidal ideations. Many experienced difficulties with fertility and childbearing and these were exacerbated in many instances by the pessimism of health care providers, resulting in confusion and depression. Physical disfigurements resulting from lupus-associated alopecia and rashes and corticosteroid-induced weight fluctuations were a major concern. These changes often affected self-image and libido, leading to strained personal relationships. Coping mechanisms that participants adopted included intense spiritual beliefs, ‘pushing through the difficult times’ and use of alternative therapies to relief symptoms was common. A poor understanding of SLE on the part of participant’s family and the community, coupled with the unpredictable course of the disease, exacerbated frustration and social exclusion. For most, limited income, lack of basic services, family dependencies, and comorbid diseases, such as human immune deficiency virus (HIV), exacerbated the daily negative SLE experiences.

**Conclusion:**

In this study of mainly indigent South African women, SLE is associated with complex, chronic and challenging life experiences. The chronic relapsing and unpredictable nature of the disease, poor understanding and acceptance of SLE, compounded by a background of poverty, inadequate social support structures, negatively impact on a range of personal, social and vocational daily life experiences. Improved access to psychosocial services and SLE education might result in better outcomes.

**Trial registration:**

(Ethics Project identification code: 275/2016 and M160633 registered 10 & 29 August 2016).

## Background

Systemic lupus erythematosus (SLE) is a chronic, multi-system autoimmune disorder predominantly affecting young women. This disease normally affects a broad spectrum of organs, most commonly the skin and mucous membranes, joints, kidneys, blood and the central nervous system [1, 2]. The disease is associated with reduced life expectancy and high morbidity, related to both active disease and therapies used to treat the disease. The chronic relapsing and unpredictable nature of the disease results in significant unmet needs affecting various spheres of health-related quality of life (HRQoL). Pain, fatigue and adverse effects of medications impact negatively social functioning, mental health and moreover complex in the case of fertility and pregnancy [3–7].

The World Health Organisation (WHO) defines HRQoL as a “multi-dimensional aspect in individuals which involves their experiences and perceptions of physical, psycho-social and mental states” [8]. HRQoL is a function of both the personal and socio-economic support structures in which the individual lives and the interaction of these with the context in which a person lives resulting in good functioning or limited functioning (disability) which in turn affects HRQoL. This is the conceptualization of functioning represented by the World Health Organization’s International Classification of Functioning, Disability and Health (ICF) (2001) [9]. This is a useful framework that could describe and understand the lives of SLE women.

SLE occurs world-wide, but for decades was reported as rare in Africa but common and severe in individuals of African ancestry living in Europe, America and Asia [10, 11]. Diagnostic delays due to poor access to health care, low awareness and limited number of specialist physicians are explanations for the underestimated prevalence of the disease on the African continent itself [12]. Data on HRQoL are scarce in developing countries, including much of the African continent [13, 14]. Poverty is an important predictor of poor outcome in any chronic illness [5, 6]. Patients living with SLE in the Low and Middle Income Countries have a lower survival rates than those living in High Income Countries [15]. The factors that contribute to this higher mortality are late diagnosis, delayed or poor intervention and infectious co-morbidities [16]. A recent narrative review of HRQoL in developing countries included 31 studies and highlighted that adverse SLE outcomes are exacerbated by poor socio-economic contexts which affect physical and mental well-being [17].

Given the lack of research on HRQoL of women living with SLE in Africa, we undertook a qualitative study by interviewing individual women with SLE to explore their “lived experience” of SLE as a chronic illness. The study was based on the phenomenology approach. We aimed to obtain understanding of issues that may be under-explored or misunderstood in routine clinical care.

## Methods

Participants were enrolled from two tertiary academic centers - Groote Schuur Hospital, Cape Town and Chris Hani Baragwanath Academic Hospital (CHBAH), Soweto. Participants from the Cape Town site were identified from the African Genetics Lupus Network (ALUGEN), a prospective registry of SLE [18]. Patients from CHBAH were recruited from the Lupus Clinic. The study was approved by the Human Research Ethics Committee at the University of Cape Town and the University of Witwatersrand.

Participants were purposively sampled to include younger (< 30 years) and older (> 30 years) age groups, those with skin involvement, fertility issues, and those with high disease activity versus those with quiescent disease, and participants with poor adherence to treatment. The study was conducted from September 2016–February 2017. All participants met the 2010 Systemic Lupus International Collaborating Clinics (SLICC) classification criteria for SLE [19], were females of 18 years or older and signed informed consent to participate. Socio-demographics including self-reported ethnic background, and disease features were documented before the individual interviews were conducted. Lupus disease activity was determined using the Physician Global Assessment (PGA) score from the Systemic Lupus Erythematosus Disease Activity Index (SLEDAI) [20].

An interview guide reflecting the main themes of pain, fatigue, emotional, social and work function, fertility, aesthetic concerns, coping mechanisms and medication adherence was developed to facilitate the process. This was based on the general issues faced by SLE women reported in other literature that we wanted to explore further. The interview process was based on the phenomenology approach to explore women’s “lived experience” of SLE as a chronic illness and in addition to give the researcher a clear and in-depth understanding of this phenomena [21, 22]. A pilot study of this interview guide was tested on four participants which were later included in the analysis. The transcripts were discussed, and adjustments were done where necessary by all authors.

Interviews were conducted in a language selected by the participant: English, Xhosa, Zulu, Tswana, Sotho, and Pedi. The interviewer and first author (AP) is a registered nurse and midwife fluent in all the languages. Interviews were conducted in a private, neutral room of the respective outpatient clinics, where noise and intrusion were avoided. Though most participants expressed the desire to be known, rather than to remain anonymous, in light of how much they suffer in silence, there was an agreement to choose a pseudonym and these have been used throughout the text. The interviewer was not at any stage involved in the care of participants.

All interviews were audio recorded, had an average duration of 70 min and were transcribed and translated into English. In addition, field notes were taken particularly on the individual each participant’s body language. No repeat interviews were done. Data saturation was reached by the 20th participant, but after discussion between the authors, five more participants were included to consolidate the existing themes.

### Data analysis

The project was set up on NVivo 11 software, and themes were identified during the pilot phase, data collection and processing [21, 22]. All participants were given individual feedback after the interview and were referred for further services if required. Thematic analysis with co-authors by going through a number of transcripts to ensure validity of the coding was done. To control for bias and data trustworthiness a number of measures were implemented. Firstly, at the time of the interview clinical data were available, and these were used to cross check critical medical information provided by informants. Secondly, the interviewer took field notes including on body language and these were compared with themes that came up from the interviews. Thirdly, to ensure consistency, Participants were asked to clarify some concepts or explain what was not clear to the interviewer. In addition, the interviewer summarised the main themes arising from participants’ narrative at the end of the interview which they either agreed with, corrected or re-worded. Fourthly, during the analysis the themes were continuously compared and were discussed early in the analysis process with all the authors to make sure that they reflected what the participants were expressing as described further below.

AP was involved in the translation process. Interviews needing translation were a mix of native languages and English in one recording, a typical communication strategy in the South African context. An accuracy check on every translation through repeated listening to the recorded interview was done while re-reading the transcription and translation and any errors rectified. AP was close to the data and the nuances required in the translation. Verbatim translation and transcription were done. AP did the initial coding using themes that emerged from the first 5 interviews. This was presented to co-authors MS and BH and they assessed the audios, translations and discussed the coding. Codes were further reviewed by all authors where it was decided that a further 5 participants were to be recruited to consolidate the existing themes. Without any disagreements, pointers on probing strategies and more codes were discovered.

The Consolidated criteria for Reporting Qualitative research (COREQ) [23] was used to prepare this paper.

## Results

Twenty-five consenting participants were interviewed. Their mean age was 30.9 years (range: 22–45) and mean disease duration was ≥5 years (range 1-5 years). Most were black Africans (72%), the remaining were of mixed racial ancestry (Table 1). Eighteen were single, 3 separated/divorced and 4 were married. Ten participants completed secondary school of 12 years, and the remainder stopped school below secondary level (5), were still at college (5) or completed formal training (5). Six participants were employed, 2 were students and the remaining were unemployed. Only 6 were recipients of state disability grants. All participants had arthritis as a presenting feature of SLE and about a quarter each had skin lesions or lupus nephritis. Eight participants were assessed to have active disease by the PGA score, and the mean score was 1.9 (range 0–4).

**Table 1:** Summary of the 25-participant’s socio-demographics, clinical features and themes explored

	*n* = 25	%
Age (years)		
> 30	15	60
< 30	10	40
Ethnicity (self-reported)		
Mixed Ancestry	7	28
Black African	18	72
Educational level		
Below Secondary level	5	20
Secondary level	10	40
At college/university/job training	5	20
Completed college/university/job training	5	20
Job status		
Employed	6	24
Unemployed	17	68
Student	2	8
Disability grant recipients	6	24
Marital status		
Married	4	16
Separated	3	12
Single	18	72
SLE features		
Lupus nephritis	6	24
Skin/discoid lupus	6	24
Arthritis	25	100
Disease Activity^a^		
High disease activity	8	32
Quiescent disease	17	68
Themes explored		
Physical Impairment:		
Pain	19	76
Fatigue	17	68
Emotional Health	25	100
Employment	25	100
Social functioning	8	32
Sexuality	22	88
Aesthetic concerns	20	80
Fertility issues	14	56
Adherence to treatment	11	44
Coping mechanisms	21	84

^a^The Physicians Global assessment of SLEDAI is a score of 1 to 4 in categories of 0 = No activity, 1 = Mild, 2 = Moderate, 3 = Severe [20]

The major themes arising from the interviews are detailed below:

### Body function

#### Physical impairments

##### Pain

Living with pain was the most complex and consistent grievance, and nineteen participants expressed living with pain almost every day. Pain was cited as the cause of “constant bad days”. The source of pain was from arthritis, and skin lesions, and 3 participants explained that they did not understand the origin of their pain. They slept on a stack of pillows or sitting position while 1 participant admitted to using large quantities of painkillers. Joint pain frequently restricted women from doing their activities of daily living.

*It [pain] is on my hands and knees mostly, sometimes I can’t even dry out my washing. If I wash, I can’t fold my hands, like this [attempting to make fists without success]. It’s painful but then it’s swollen also (Chan; 35 years, unemployed).*

Younger participants were worried about the chronicity of pain and how it affected their ability to function, their sleep and their present and future life expectations. Several factors aggravated their pain including erratic adherence to medication, a cold environment and physical activity. The pain was often coupled with muscle stiffness especially in the mornings. Despite the pain, many still had to care for dependants.

The nature of pain was described in diverse ways and all participants expressed that they struggled to explain the nature of the pain to their families, employers, community and health care providers (HCPs). Furthermore, many felt that the concept of “pain” did not describe their experience adequately.

*It’s as if you are tied in wires around the joints! (Neelo; 25 years-old former domestic worker, unemployed due to severe arthritis).*

*It feels like somebody’s chipping away at your bones with a nail and a hammer especially when it’s cold! (Larona; 23-year-old university student).*

##### Fatigue

Seventeen participants disclosed living with fatigue which they described as “severe loss of energy” and “a strong need to sleep”. They expressed it as the most emotionally draining experience that no one, including HCPs, understood fully. Participants reported being viewed as ‘simply lazy’ by most family members. One participant expressed frustration at being told by her doctor that fatigue was “normal”. Fatigue impaired activities of daily living (ADL), including taking care of dependents and employment.

*I will not iron the laundry. I leave it. But I would wake up tired, feeling that today, I am doing nothing. I do not clean, I do nothing. I sit. I do not have strength. There is nothing I can do. I cannot even go to the shop (Tumi; 43-year old, unemployed).*

One participant described a day without fatigue as a ‘happy day’ which occurred ‘seldom’. While some participants succumbed to the feelings of fatigue, a few stood up against it.

*Even if I’m tired, I keep on doing what I’m supposed to be doing. So, I can’t really say it stopped me to do anything, as I said to you I’m strong, I’ll just force myself to do things. I feel everything is only in the mind. If you say, “Today I’m tired”, then you’ll stay in bed and you’ll stay tired the whole day. So, I’m not that kind of a person. I’ll wake up in the morning and I’ll feel tired and I’ll say, “Today I told myself that I’m going to do this,” and I’m going to do it tired or not. So, I’m that kind of a person. Even if I feel tired, still it doesn’t stop me (Kiswa; 40 years old, separated mother of three).*

One participant (37-year-old Lizzie, unemployed) described her tiredness as a feeling of being in a very long distance race. Furthermore, it was coupled with weakness and dizziness and struggling with sitting for extended periods of time. Like most participants, despite not being physically active at all, she felt as though she had just carried out a very heavy task.

Three participants struggled with fatigue in the workplace and often found themselves sleeping while on duty and feared losing their jobs. Participant Olivia expressed her gratitude for the interview opportunity as it gave her a platform to voice that fatigue is not normal.

Most participants suspected that fatigue could be from SLE and in 1 case, severe fatigue was the leading symptom that led to the diagnosis of SLE. However, most participants did not associate fatigue with disease activity, while some associated it with sun exposure, body pain or evil spirits.

One participant (Tumi; 43 years old, unemployed) blamed supernatural powers:

*On Sunday at church, I was so tired, I know that it is the spirits from there because when I arrived… mmh!... fatigue came. It’s the evil spirits! [patting her shoulders, slouching on the chair, shaking her head vigorously and displaying signs of being defeated].*

#### Emotional function

##### Poor emotional and mental health

All 25 participants expressed various kinds of emotional problems due to the SLE diagnosis and its complications. These included dealing with the anonymity of SLE, job and career loss, aesthetic concerns, struggles with fertility and pregnancy, pain, fatigue, poor memory, sexual dysfunction and loss of intimate relationships. Feelings of sadness, anger, anxiety and symptoms of depression were voiced. Most participants expressed feeling emotional ‘out of nothing’ and having extremely bad days full of negative emotions.

The most striking interview was with 25-year-old Olivia, employed, mother of one, who had several suicidal attempts without anyone’s knowledge.

*When I started, I would take ten pills, drink them. The second time when I get to that position I thought, okay, the last time I had ten pills and they didn’t work, meaning this time I must drink more than that. Afterwards, I feel weak, sleepy, sweaty, but I don’t say, and nobody knows - you are the first person, the first person to know about this. (sobbing uncontrollably).*

Only 3 participants had been diagnosed with depression. One was diagnosed with depression after being diagnosed with human immune deficiency virus (HIV) infection. She admitted to weaning herself off the anti-depressants as they made her feel out of touch with reality. In one case, the participant was adherent to her anti-depressant but showed poor coping skills while in another case, the participant stopped her medication because she believed she was not crazy. The latter participant later relapsed and needed to restart her antidepressants. One participant showed features of undiagnosed post-traumatic stress disorder (PTSD) after a miscarriage. After their interviews, 5 participants were referred, with their consent, back to their respective clinicians for further management of the depression.

### Environmental factors

#### Poor socio-economic status (education and jobs)

Most participants believed they deserved a social grant due to their body limitations, and 16 reported SLE-related joint pain together with fatigue as the cause of their unemployment. They struggled with work conditions such as cleaning, doing laundry, sitting for a prolonged time, a cold environment and carrying plates in a restaurant. Those who had resigned from their formal employment, cited challenges like SLE-related role limitations, together with employer dissatisfaction for the multiple hospital visits and admissions or being more at home than at work. All the 6 formally employed participants had completed tertiary education. Those without higher qualifications struggled to find or continue work. More than half of the participants had either left school before completing secondary education or had obtained inadequate grades and had abandoned tertiary education. For most, further education was impossible without financial means.

*Jobs are also not [there] because I am not educated, it’s not easy to get a job. When I found out I have the Lupus disease I was working as a domestic worker. That’s what affected me [job nature], because of the water I was doing the laundry with hands, not with a laundry machine. It’s so difficult. It’s hard (Neelo; 25-year-old former domestic worker, unemployed due to severe arthritis).*

*This arthritis, my wrists would get sore, one side of the wrist would get sore then I couldn’t work. Maybe I could work with one hand (Phumlani; 28-year-old previous chef).*

On a change in lifestyle, 33-year-old Sinethemba (former domestic worker and mother of two living with lupus nephritis) disclosed that she had no choice but to sit and do nothing due to the pains and could not force herself to do anything.

Limited household income was a major concern for most participants. This coupled with a poor understanding of the chronicity of SLE affected adherence to medication and attendance of follow-up visits to the Lupus clinic. Lack of transport money was also a main contributor to poor access to care amongst these indigent women.

*I am now feeling better. It’s not like in the beginning when I was very sick. I had decided to quit due to circumstances. This year I was telling her that “mom, I am quitting treatment because we don’t have money. You [mother] are supporting the family and I also require money for travel” (Neelo; 25-year-old former domestic worker, unemployed due to severe arthritis).*

#### Poor social health

The unpredictable course of the disease and limitations to physical activities forced many women not to socialize at all. They explained that the South African social system requires attendance at funerals, weddings and parties. For one participant on peritoneal dialysis for chronic renal failure, food and drinks at social events were a challenge given her dietary and fluid restrictions. In addition, these events required a lot of physical activity that most participants could not cope with.

*“You must do something madam! (imitating a third person)”, and when you are busy peeling potatoes; peel potatoes pain, peel potatoes pain, pain, pain, pain, pain; the whole night! (Selina; 24 years old, employed).*

Many participants described feelings of being drained by interacting with new people and struggling to explain their diagnosis. Hence, they resorted to being alone.

When asked about what the community understood about SLE, nine participants explained that SLE was confused with HIV, cancer, death or witchcraft.

*When I lost weight, the community said I have HIV, it was depressing! When I gained weight, they said “she’s starting to be fresh, she is on ARV’s (antiretroviral therapy)” (Trisha; 29 years old, jewellery designer trainee).*

Thuswa (37 years old, IT consultant) described the reasons for her cleansing ceremony in church as arising from the community’s comments about her illness:

*No, there’s something wrong with you! You have a demon. We [church] have to get that demon out!*

### Activity limitations

#### Poor sexual health

Problems with sexuality became one of the major themes expressed. For most, a woman’s image is her sexuality, and aesthetic concerns were a main hindrance to fulfilled intimacy as they lacked the confidence and courage to engage.

*I don’t feel that I want to be sexually active or intimate. At night I don’t sleep with a wig; with this bald head, I am shy and uncomfortable (Nino; 27 years old, unemployed, newly-wed).*

Fatigue and pain were also listed as contributors to poor sexual health causing tensions in intimate relationships. The majority of single and separated participants disclosed that poor sexual health was the main cause of their failed relationships and felt that their partners did not understand their condition.

##### Aesthetic concerns

Participants expressed distress at a range of the SLE-related or treatment-related body changes including moon-shaped face, weight gain, weight loss, alopecia, discoid skin lesions and scars. Most felt they could not dress the way they desired. The participants who were single or separated felt their single status was mostly due to aesthetic concerns, which in turn affected self-image and libido.

*…because of my body, my husband left me because he’s not attracted to me. I’m not cute enough for him anymore. So those are the things that make me feel so down (Kiswa; 40 years old, separated mother of three).*

##### Skin lesions

Living with discoid skin lesions did not cause only excruciating physical pain, but raised emotional, social and economic issues due to the change in appearances, for about a quarter of the participants. One of them was quoted saying:

*When the discoid lupus started, that’s when I stopped going out, oh my goodness they (everyone) were staring at me (Phumlani; 28 years old, previous chef).*

Perceptions on how they were seen by their society caused feelings of sadness, anger and discomfort. Most participants had low self-esteem and explained that the looks from the community or workplace colleagues were driving them to stay behind closed doors.

### Personal factors

#### Fertility concerns

Childbearing was one of the important concerns and some expressed the stresses and trauma of pregnancy and fertility experiences. Many participants were put-off by the pessimism of their HCPs regarding prospects of having a family, and cited this as a cause of confusion and depression.

*Two years, no child. Some of them [in-laws] are going to say, “You must take this one out, this is a barren [woman]” (28-year-old Zayo, unemployed).*

*The doctor is not God, so he can’t say that. I want to experience to be a mother. He said I won’t be able to have children and I didn’t believe him. I prayed and now I have two. They told me the children won’t be normal. Both are normal (Chan; 35 years old, unemployed).*

#### Adherence to treatment issues

Eleven participants admitted to poor adherence to medication. The reasons they offered included the overwhelming number of tablets they were expected to take every day, a belief that they were not sick especially when the illness was inactive, forgetfulness and side effects of the medications such as weight gain, unpleasant taste, nausea, and loss of a sense of reality (felt to be due to antidepressant medication):

One participant blamed her lupus flares on the tablets:

*Too many tablets are not good, they all clash with one another. I even stopped the anti-depressant because I am not mentally sick. I’m full of side effects, that’s why I’m so sick (Inn; 40 years old, unemployed).*

While most participants had branded chloroquine “the bitter pill” as it has a bitter taste when swallowing it, they felt that their lives were bitter just like the taste of the chloroquine.

Despite their reluctance to take many tablets, lupus flares following poor adherences had forced most participants to take all their medication, making use of daily pill boxes or reminders from a phone alarm or family member.

#### Participants understanding of SLE

Twenty participants had a reasonably good understanding of SLE, while five could neither explain nor accept the diagnosis:

*I don’t get what Lupus is, I just take my medications and come for check-ups (Neelo; 25-year-old former domestic worker, unemployed due to severe arthritis).*

Many found the disease mysterious and unpredictable, often with a malicious intent, a silent killer that has been there for years. Some described the SLE outcomes, especially the excruciating pain, as something they would not wish for anyone, even their worst enemy.

*Lupus is a very sneaky disease. It attacks you at a time that you are dumbstruck. It just slaps you in your face and you’re down. I don’t know what causes the flare-ups or what triggers it (Debbie; 43-year-old teacher).*

*A disease that one would never really know it until one gets it, one would never know what pain and tiredness is until they get it; an irritating and frustrating disease (Tina; 29-year-old call center employee).*

*It’s not like AIDS (acquired immune deficiency syndrome) - this one is still undercover. “We only know about it when we are here at the clinics” (Selina; 24 years old, employed).*

### Coping strategies

#### Family support and alternative medicine

Despite most participants describing problems socializing, family support was one of the most important tools that helped them cope with SLE. All participants reported that having either a family member, friends or partner support gave them strength to cope with the unpredictable nature of the disease. Some revealed that their supporters went as far as arranging alternative medicines for them. Moreover, participants felt that patient-led support groups, better education by HCPs, and public awareness of SLE would help them cope better with the disease. Two participants explained that their medications consistently helped prevent fatigue. Strong disease acceptance and the drive to care for oneself or a dependant was helpful. Some described their feelings as follows:

*“I hold my head high and I have a reason to live; my young son- he needs me” (Tina; 29-year-old, call center employee).*

*I’m a strong woman, I’ve fought with HIV to hell and back, I’m going to beat this SLE. I’m going to take medication as I’m doing with my HIV medication and I’m going to look after myself as I always did (Kiswa; 40 years old, separated mother of three).*

Many participants explained that alternative therapies helped contain the symptoms of SLE. These included drinking ‘blessed’ water from church, strong spiritual beliefs in God, traditional herbal remedies, and soothing elephant tree leaves or pressure bandage applications to painful joints.

**Fig. 1:**
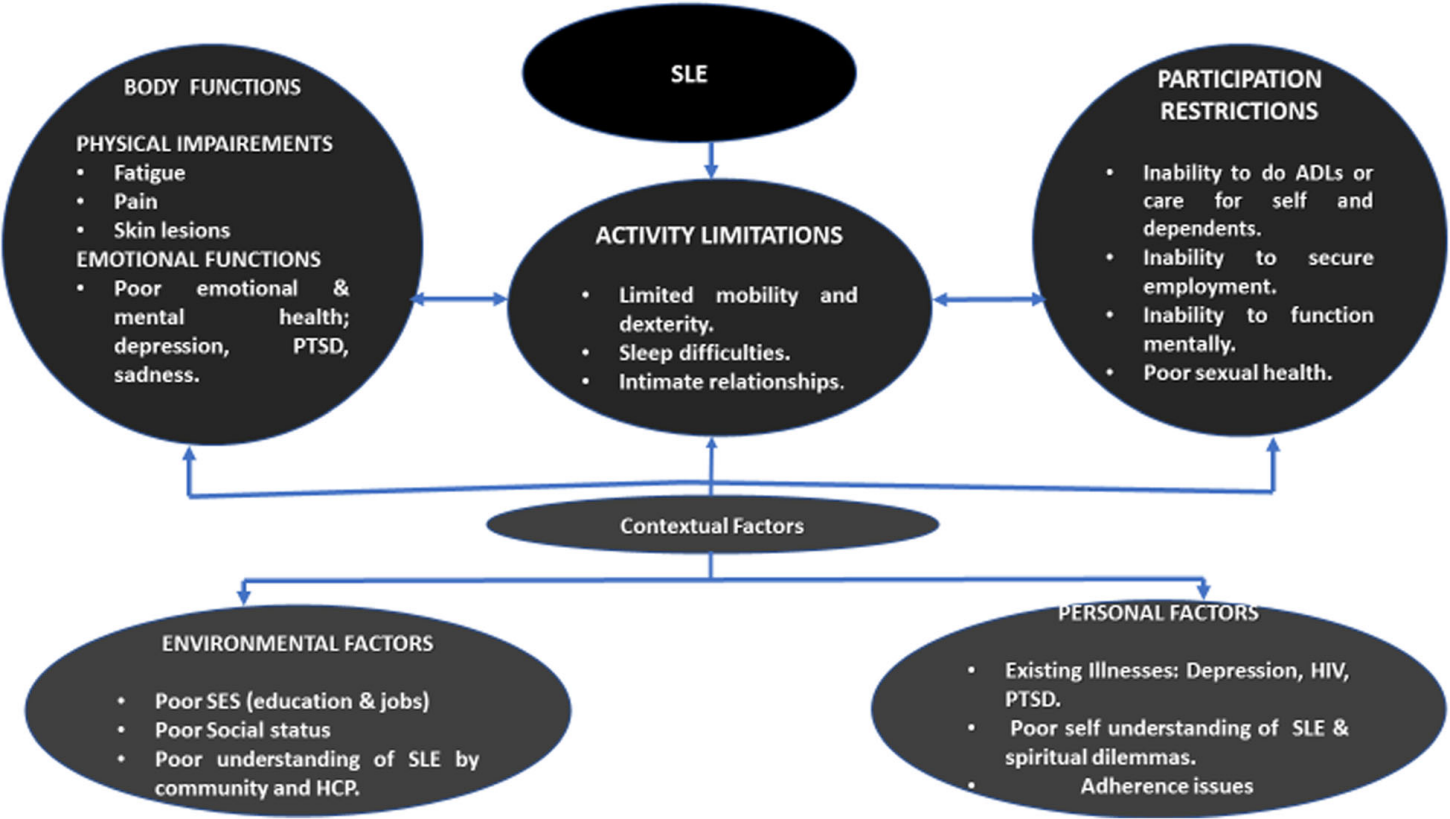
Interactions between experiences of SLE women and their general health outcome using the International Classification of Functioning, Disability and Health (ICF) framework. Abbreviations: *ADL*: activities of daily living, *HIV*: Human immune deficiency virus, *HCP*: Health care provider, *PTSD*: Post traumatic stress disorder, *SES*: socio-economic status, *SLE*: Systemic lupus erythematosus

The three levels of functioning in Fig. 1 interrelate, are intertwined with each other and are affected and influenced by the contextual factors of a patient living with SLE. The individual body structure and function impairments (pain, skin lesions, fatigue and existing illnesses such as HIV, depression, and post-traumatic stress disorder) negatively affected activities executed by the participants, specifically limited mobility and dexterity, sleep and engaging in intimate relationships. The activity limitations interacting with the external environmental factors and personal factors resulted in difficulties in caring for themselves or dependants, carrying out household tasks, securing employment, functioning effectively mentally or having intimate relationships. The environmental and personal factors that interacted with the activity limitations included poor economic status (education and jobs), poor social status, poor understanding of SLE by community and HCPs**,** poor self-understanding of SLE, and existing illnesses and spiritual dilemmas and adherence issues.

## Discussion

This qualitative study reflects on the challenges of women living with SLE in poorly resourced communities of South Africa. We explored activity limitations that were exacerbated by lack of understanding and negative attitudes of family, friends and employers, costs of transport, and other factors resulting in restricted participation in work, social life and intimate relationships. Women living with SLE reported on some of the obstacles and factors which may negatively impact their HRQOL. The ICF framework demonstrates a level of disability which is an outcome of complex interactions between the SLE, activity limitations and contextual factors as shown in Fig. 1 [8, 9]. Strikingly, SLE remained mysterious to the participants and their communities, and this poor understanding exacerbated frustration and life uncertainties.

The confusion of HIV with lupus was a common experience amongst the participants. This is not a surprising finding as the symptoms of the two illnesses overlap [24]. In the South African context both illnesses affect mainly young women and the overlapping features of the two conditions not infrequently lead to either in misdiagnosis or delay in diagnosis of SLE. In our study, living with both these diagnoses caused major distress amongst the women.

Our study shows that SLE has a severe impact on social lives resulting in self-imposed isolation from society at large, and impaired sexual health leading to either separation or singlehood status. These findings are similar to those described amongst Ecuadorian women with SLE were the majority of young women expressed unhappiness at not being married [25].

Two previous South African studies have explored HRQoL - a quantitative study showed poorer social functioning amongst SLE compared to rheumatoid arthritis (RA) patients [14], and a qualitative study in RA highlighted social exclusion and lack of independence exacerbated by poverty [26]. In our study aesthetic concerns dominated the narratives on how it affected self-esteem and, in turn, affected both social participation, intimate relationships and work opportunities.

Family support was a key element that assisted participants to cope with the disease. Their responsibility towards their dependants and conversely, family members offering support, gave them greater strength to cope with the illness. Moreover, spiritual beliefs were an important component of the coping strategies for many participants. Two Brazilian quantitative studies have similarly highlighted that SLE patients with strong spiritual beliefs had a positive outlook on life and coped better with the diagnosis [27, 28]. Unlike the participants in a study from the United Kingdom who expressed some positive aspects of living with SLE [29], none of the women in our study felt upbeat about their diagnosis.

Much work is needed in developing and testing of local coping strategies to overcome pain, fatigue and mental health challenges associated with SLE. These might include patient-led support groups and improved access to psychosocial services. Ongoing training for HCPs together with community awareness programmes might result in better understanding of SLE. In addition, considering the high rate of unemployment and lack of formal training amongst these women, better disability support and training opportunities are vital to alleviate the poverty that complicates the lives of patients with SLE.

Whilst our results are a good reflection of the experiences of women with SLE attending the tertiary level facilities, unknown biases could have arisen during the analysis process. As the themes were presented by the first author to the co-authors for discussion, the themes were further refined. During this process, some important themes might have been excluded. In addition, variations in the moods of participants at the time of interview, might have affected their responses.

## Conclusion

In conclusion, notwithstanding these limitations, our study highlights the many complex, chronic and challenging life experiences of indigent South African women with SLE. A poor understanding, perception and acceptance of SLE by both patients and the community at large, coupled with unpredictable and mysterious nature of the disease has a profound negative impact on multiple dimensions of SLE patients. Physical disability due to pain and fatigue together with aesthetic concerns have a strong negative effect impact on mental health, social functioning, job acquisition and sexual health. Family support and spirituality are major coping strategies but stigma and lack of understanding by others also negatively affects their participation in major life activities such as employment. As mentioned above, comprehensive, multi-pronged approaches are likely to improve the overall lived experience of women with SLE.

## Abbreviations

ADL: Activities of daily living
AIDS: Acquired immune deficiency syndrome
ALUGEN: African Genetics Lupus Network
ARV’s: Antiretroviral therapy
COREQ: Consolidated criteria for Reporting Qualitative research
HCPs: Health care providers
HIV: Human immune deficiency virus
HRQoL: Health-related quality of life
ICF: International Classification of Functioning, Disability and Health
PGA: Physician Global Assessment
PTSD: Post-traumatic stress disorder
RA: Rheumatoid arthritis
SES: Socio-economic status
SLE: Systemic lupus erythematosus
SLEDAI: Systemic Lupus Erythematosus Disease Activity Index
SLICC: Systemic Lupus International Collaborating Clinics
WHO: World Health Organisation
WHOQoL: World Health Organisation Quality of Life
